# Immunomodulatory effect of *Tinospora cordifolia* extract in human immuno-deficiency virus positive patients

**DOI:** 10.4103/0253-7613.42302

**Published:** 2008-06

**Authors:** M.V. Kalikar, V.R. Thawani, U.K. Varadpande, S.D. Sontakke, R.P. Singh, R.K. Khiyani

**Affiliations:** Department of Pharmacology, Government Medical College, Nagpur - 440 003, India; 1 Department of Preventive and Social Medicine, Government Medical College, Nagpur - 440 003, India; 2 Department of Skin and Venereal Diseases, Government Medical College, Nagpur - 440 003, India; 3 Department of Rasa Shastra, Government Ayurvedic College, Nagpur- 440 009, India

**Keywords:** CD4 count, herbal extract

## Abstract

**Objectives:**

To assess the safety and efficacy of TCE in human immuno-deficiency virus positive patients.

**Materials and Methods:**

Efficacy of *Tinospora cordifolia* extract (TCE) in HIV positive patients was assessed in randomized double blind placebo controlled trial. 68 HIV positive participants were randomly assigned to two groups to receive either TCE or placebo for six months. After clinical examination TLC, DLC, ESR, platelet count, hemoglobin and CD4 count were done. The hematological investigations were repeated at bimonthly intervals and CD4 count was repeated at the end of the study. Patients were clinically reviewed at monthly intervals for compliance, refill and ADR monitoring. The drugs were decoded at the end of the trial.

**Results:**

TCE treatment caused significant reduction in eosinophil count and hemoglobin percentage. 60% patients receiving TCE and 20% on placebo reported decrease in the incidence of various symptoms associated with disease. Some of the common complaints reported by patients on TCE were anorexia, nausea, vomiting and weakness.

**Conclusion:**

*Tinospora cordifolia* extract, a plant derived immunostimulant, significantly affected the symptoms of HIV. This was validated by clinical evaluation. However not all of the objective parameters studied by us, back this up. *Tinospora cordifolia* could be used as an adjunct to HIV/AIDS management.

## Introduction

Acquired immuno-deficiency syndrome (AIDS) is a fatal illness caused by human immuno-deficiency virus (HIV), which breaks down the host immune system, leaving the subject vulnerable to life-threatening opportunistic infections, neurological disorders and malignancies. Once infected, it is probable that a person will be infected for life. In 2001, 40 million people were living with HIV/AIDS worldwide, prevalence rate was 1.2%, about 5 million became infected and about 3 million died of it.[1] With high fatality, significant impact on quality of life, lack of curative treatment or vaccine, HIV/AIDS has become a serious global health problem.

The main cause of immune defect in AIDS is deficiency of the thymus-derived lymphocytes (T_4_), characterized by the presence of CD_4_ surface molecules, which are the cellular receptors for HIV.[2] Anti-retroviral therapy (ART) has issues associated with availability, affordability, toxicity, ADR monitoring and emerging resistance. In the absence of effective cure, immunorestorative therapy seems logical adjunct. It has been suggested that when routinely used pharmacological interventions fail to relieve the symptoms, immunotherapy may be effective.[3] It is therefore worth looking for a natural product that is safer, affordable, effective, better tolerated and devoid of side effects.

*Tinospora cordifolia* (Wild) Miers is a deciduous climbing shrub indigenous to tropical Indian subcontinent, belonging to the family Menispermaceae. In Ayurveda *Tinospora cordifolia* (TC) is used as “*rasayana*” which has powerful immunostimulant activity.[4] Charaka described rasayana as antiaging, which increased the life span, promoted intelligence, improved memory and ensured freedom from diseases, indicating immunostimulant effect.[5]

Pretreatment with TC afforded protection against induced infections in mice[6] and rats.[7] In patients with obstructive jaundice, addition of TC to routine surgical procedure increased the survival rate and also increased polymorphonuclear leukocyte functions suggesting an increase in phagocytosis and intracellular killing capacity.[8] TC extract (TCE) in the same dose as used in this study, showed significant beneficial effect in allergic rhinitis[9,10] and burns.[11] Hence this study was planned to scientifically assess its efficacy in HIV positive patients.

## Materials and Methods

This was a prospective, randomized, double blind, placebo controlled comparative trial carried out in the AIDS counseling center of Skin and Venereal diseases department of Government Medical College Hospital, Nagpur with permission from Institutional Ethics Committee. Patients diagnosed to be HIV positive were approached with the request to participate in this trial. Those who showed interest were given detailed information handouts about the trial, in the language understood by them. From those who volunteered to participate, informed witnessed written consent was taken. The inclusion and exclusion criteria were:

**Inclusion criteria**
Subjects diagnosed to be HIV positive
Attending the HIV counseling center of GMCHN
Volunteering to participate and give informed written consent
Of either gender in the age group of 18 - 50 years.

**Exclusion criteria**
Patients on ART
Taking immunomodulators
Suffering from associated diseases like hypertension, ischemic heart disease, diabetes, or CNS disorders.
Clinical evidence of end organ damage
Pregnant or lactating women
Participation in any medicine trial in previous one month.

Baseline pulse, respiratory rate, blood pressure and temperature were recorded. After clinical examination, 68 participants were entered in the trial. They were randomly assigned to two groups of 34 each to receive either coded TCE 300 mg per tablet or matching placebo in the same formulation, packing, size, weight, color and dose of one tablet three times a day for six months. Baseline TLC, DLC, ESR, platelet count, hemoglobin and CD_4_ count were done.

The medicines were issued to the participants for one month at a time and refill was given during monthly recall. During recall the participants were asked to bring the leftover medicines along with the container. 80% drug consumption was considered to be compliant. All the returned medicines were discarded. At each monthly visit the patients were asked to report the occurrence of fever, cough, easy fatigability, arthralgia, oral ulcers, diarrhea, skin rash or any other symptom. The participants were at liberty to withdraw from the trial at any time. The participants were explained to take treatment whenever necessary, for minor complaints like fever, diarrhea, skin diseases, pain and also for other infections like tuberculosis.

The TLC, DLC, ESR, platelet count, Hb% were repeated at bimonthly intervals. CD_4_ count was repeated at the end of the study. The trial medicines were decoded at the end of the trial and the participants were followed up for additional one month for monitoring the residual effects, if any. Institutional ADR monitoring and reporting system was followed. All data was logged in the case record form by principal investigator. The results were analyzed by Kruskal Wallis test.

M/s Pharmanza India Ltd, Gujarat, India supplied the TCE (Tinofend ^R^) and matching placebo, free of cost, on our request. Each tablet of Tinofend contained 300 mg of standardized extract obtained from water extract of stem of TC. It contained more than 5% bitter principles and was tested for presence of cordioside and tinosporoside by HPLC.

## Results

In study participants, HIV was more common in the 18-30 years age group. Half of the enrolled patients were from this age group, 42% from 30-40 years, and 8% from 40-50 years age group. The men: women ratio was 1: 1.06. All had contacted the HIV infection through sexual contact. None of the participants was commercial sex worker. Among the participants were 9 couples. The family history of the participants revealed that spouses of 15 participants (not included in this study) were infected with HIV/AIDS.

Kruskal Wallis H test was used for determining statistical significance. There was no significant difference in the baseline parameters between the two treatment groups [Table 1].

**Table 1:** Comparison of baseline characteristics of two treatment groups *(Mean ± SD)*

*Parameters*	*Intervention*	
	
	*Tinospora cordifolia*	*Placebo*
CD4	289.5 ± 200.3	282.9 ± 214.1
TLC	6309.7 ± 2022.0	6910.0 ± 3413.5
Neutrophils	60.5 ± 10.2	63.8 ± 8.7
Lymphocytes	30.0 ± 9.2	27.9 ± 8.7
Eosinophils	5.0 ± 2.2	4.8 ± 2.8
Monocytes	3.9 ± 1.8	3.4 ± 1.4
ESR	16.9 ± 6.2	18.1 ± 8.9
Platelets	2.3 ± 0.7	2.4 ± 0.9
Hemoglobin	12.6 ± 1.3	12.0 ± 2.2

In participants who received TCE, a significant reduction in Hb% and eosinophil count was seen after 6 months of treatment. Statistically significant difference was not seen in other studied parameters [Table 2].

**Table 2:** Comparison of parameters before and after Tinospora treatment (Mean±SD)

*Parameters*	*At baseline*	*After 6 months*
CD4	289.5 ± 200.3	296.2 ± 195.2
TLC	6309.7 ± 2022.0	5647.3 ± 1715.0
Neutrophils	60.5 ± 10.2	60.6 ± 8.9
Lymphocytes	30.0 ± 9.2	32.7 ± 8.9
Eosinophils	5.0 ± 2.2	3.5 ± 1.2*
Monocytes	3.9 ± 1.8	3.9 ± 1.5
ESR	16.9 ± 6.2	17.3 ± 5.1
Platelets	2.3 ± 0.7	2.0 ± 0.4
Hemoglobin	12.6 ± 1.3	11.8 ± 1.3*

(* *P*<0.05 when compared to baseline)

Among the participants who received placebo, there was no difference in CD4 count before and after six months. There was statistically significant reduction in TLC, neutrophil and eosinophil count. The mean lymphocyte count at the end of treatment was significantly higher as compared to baseline [Table 3]. No statistically significant difference was noted between the two groups [Table 4].

**Table 3:** Comparison of parameters in placebo group before and after treatment (Mean±SD)

*Parameters*	*At baseline*	*After 6 months*
CD4	282.9 ± 214.1	268.0 ± 205.3
TLC	6910.0 ± 3413.5	5433.8 ± 1766.1*
Neutrophils	63.8 ± 8.7	58.4 ± 10.1*
Lymphocytes	27.9 ± 8.7	32.6 ± 6.9*
Eosinophils	4.8 ± 2.8	3.1 ± 1.4*
Monocytes	3.4 ± 1.4	3.2 ± 1.2
ESR	18.1 ± 8.9	19.1 ± 7.7
Platelets	2.4 ± 0.9	2.0 ± 0.5
Hemoglobin	12.0 ± 2.2	11.7 ± 1.6

* *P*<0.05 when compared to baseline

**Table 4:** Comparison between the two treatment groups after treatment (Mean±SD)

*Parameters*	*Intervention*	
	
	*Tinospora cordifolia*	*Placebo*
CD4	296.2 ± 195.2	268.0 ± 205.3
TLC	5647.3 ± 1715.0	5433.8 ± 1766.1
Neutrophils	60.6 ± 8.9	58.1 ± 10.1
Lymphocytes	32.7 ± 8.9	32.6 ± 6.9
Eosinophils	3.5 ± 1.2	3.1 ± 1.4
Monocytes	3.9 ± 1.5	3.2 ± 1.2
ESR	17.3 ± 5.1	19.1 ± 7.7
Platelets	2.0 ± 0.4	2.0 ± 0.5
Hemoglobin	11.8 ± 1.36	11.7 ± 1.6

During the monthly recalls, participants were clinically examined and evaluated. From the TCE treated group, 60% reported decrease in symptoms, 30% reported no change and 10% patients reported deterioration. On the other hand in the placebo group, 40% patients reported an increase in symptoms, 40% patients reported no change and 20% patients reported decrease in symptoms.

Out of 68 participants entered in the trial, 8 dropped out. On decoding, 5 of them were found to be on placebo and 3 on TCE. Those on placebo did not wish to continue because of lack of relief from symptoms. Those on TCE dropped out due to complaints of anorexia, nausea, vomiting and weakness. One patient receiving TCE died during the trial.

## Discussion

Pharmaceutical companies like Merind, Himalaya, Krauter market TC in India, either singly or in combination with other herbs, and all recommend these formulations for immunostimulant action. However we could not find any report relating to TC in HIV/ AIDS patients. Hence we lack the data of other studies for comparison.

Other studies have reported that mice treated with TC showed significantly greater phagocytic and intercellular bactericidal capacity of polymorphs.[6] TCE stimulated peritoneal macrophage in a dose-dependent manner.[12] TC caused increase in phagocytosis and intracellular killing capacity as suggested by the increase in survival rate and polymorphonuclear leucocyte function when TC was added to routine surgical procedure in patients with obstructive jaundice.[8] In patients of allergic rhinitis, TCE improved the symptoms of nasal discharge, sneezing, nasal pruritus and nasal obstruction and increase the total leucocyte count.[9] With such illustrious efficacy reports we expected TCE to show some action in HIV/AIDS. As one of the prime parameters for this we used the CD4 count on which TCE did not show any significant effect.

In our study there was significant reduction in the leukocyte count in participants receiving placebo, while in those receiving TCE there was no significant reduction. The decrease in TLC in placebo group may be due to the natural course of the disease, while in TCE group, the fall in leukocyte count, may have been checked by virtue of its “rasayana” effect. We also noted significant reduction in the eosinophil count with six-month treatment with TCE. Reduction in eosinophils has been reported with immunotherapy.[13] This supports the immunomodulatory action of TCE.

It has been reported that TCE is also capable of stimulating B-lymphocytes, macrophages and polymorphonuclear leucocytes.[14] But in our study the mean lymphocyte count in patients receiving TCE did not change significantly, while in the placebo group it showed significant increase, for which we do not have explanation.

Even though we did not get appreciable positive results with TCE on the hematological parameters, the patients on TCE reported significant symptomatic improvement, which correlated with clinical evaluation. This was evident from the requests from most of the participants of TCE group to continue providing them TCE even after conclusion of the trial. However we do realize the limitation of scientific validity of subjective benefits.

Three out of the 34 patients on TCE complained of ADRs like anorexia, nausea, vomiting. In the rest TCE was well tolerated. The patient who died during trial was on TCE. His CD4 count was 245 at baseline. There is no conclusive evidence that his death was as a result of intervention or natural course of the disease. It is known that HIV infection progresses at a very slow pace and may take nearly a decade for full-blown AIDS to develop.

Not all research results in positive findings and this one unfortunately is among those. We feel that six months may not have been enough for studying the efficacy of TCE on HIV positive patients.
